# Personality profile of individual sports champions

**DOI:** 10.1002/brb3.2145

**Published:** 2021-05-05

**Authors:** Paweł Piepiora

**Affiliations:** ^1^ Department of Sports Didactics Faculty of Physical Education and Sports University School of Physical Education in Wrocław Wrocław Poland

**Keywords:** behavioral psychology, championship, NEO—five‐factor inventory, personality, psychology, sport psychology

## Abstract

**Background:**

Research on personality in sport is popular because it allows you to forecast the greediness of actions in sports competition situations. The purpose of this paper is to determine which personality traits characterize individual sports champions.

**Materials and Methods:**

The subjects of the research were Polish athletes (*N* = 600) between 20 and 29 years of age from 20 individual sports disciplines (each *n* = 30). Then, a sample of champions (*n* = 56) and other individual disciplines athletes (*n* = 544) was selected from the study population. The Big Five model was used to examine their personality (NEO‐FFI).

**Results:**

Individual sports champions were characterized by a lower level of neuroticism, a higher level of extraversion, agreeableness, and conscientiousness in relation to other athletes.

**Conclusion:**

Each sports discipline is characterized by slightly different psychological requirements for athletes. The undertaken sports activity shapes the personality, and the shaped personality traits have an impact on taking solutions in the starting situation. The level of intensity of neuroticism, extraversion, agreeableness, and conscientiousness may determine the result in competition in individual sports.

## Graphical abstract

The purpose of this paper is to determine which personality traits characterize individual sports champions. Sports activity shapes the personality, and the shaped personality traits have an impact on taking solutions in the starting situation. The level of intensity of neuroticism, extraversion, agreeableness, and conscientiousness may determine the result in competition in individual sports.

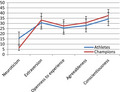

## 1 INTRODUCTION

In the field of physical culture, it is well known what the physical determinants of success in sport are. They are determined by somatic predispositions, as well as motor, technical, and tactical preparation. Somatic predispositions are mainly genetically determined and are verified at the stage of sports selection, while physical preparation is determined by the specificity of the trained sports discipline. These physical conditions are achieved through well‐organized sports training. It might seem that this is a recipe for success. But despite somatic predispositions and physical conditions, most athletes cannot achieve championship. There are many training masters but only a few champions. Therefore, it is assumed that the psyche is behind the success of a physically well‐prepared competitor. Hence, the psychological determinants of success in sport have become the interest of sports psychologists. Just as the physical determinants of success in sport are divided into four components, the mental determinants of eudaimonia in competition can include personality factors, temperamental factors, agitation control, self‐confidence, mental resilience, concentration, relaxation, and others. But the greatest importance is given to personality (Allen et al., 2013; Allen & Laborde, 2014; Kang et al., 2016; Piepiora, 2020).

The general profile of athletes in terms of the Big Five five‐factor personality model (McCrae & Costa, 2003) is low neuroticism, high extroversion and conscientiousness, and average openness to experience and agreeableness (Fuller, 2011; Mirzaei et al., 2013; Paunonen, 2003; Piedmont et al., 1999; Piepiora & Kaśków, 2019; Piepiora et al., 2018; Shrivastaval et al., 2010; Watson & Pulford, 2004). On the other hand, it is extremely difficult to distinguish and define the most favorable type of personality, as it is largely influenced by the trained sports discipline and personality conditions of the athletes depend on it. In sports theory (Piepiora et al., 2020), three sports groups are distinguished: individual sports, team sports, and combat sports. The aim of this paper is to define which personality traits characterize individual sports champions. Generally, the personality dimensions that significantly differentiate the sports level of players in individual disciplines include neuroticism, extraversion, and openness to experience (Allen et al., 2011). However, recent studies of personality in sport have shown a lack of clarity. Against the background of the abovementioned personality traits, Piepiora and Witkowski (2020b) demonstrated low neuroticism as the main mental factor distinguishing sports champions from the rest of the players, allowing for effective actions in difficult situations, whereas Allen et al., (2021) in their review, attributed this significance to extraversion. In their opinion, it is extraversion that significantly differentiates sports groups. In addition, highly extroverted athletes employ more adaptive action strategies, have stronger coach–player relationships, and tend to be more successful. Therefore, the intention of this study was to define personality determinants of success in individual sports.

## 2 MATERIAL AND METHOD

### 2.1 Research group

The research was carried out between October 1, 2015, and September 30, 2019. The subjects of the research were athletes (*N* = 600) from 20 individual disciplines, selected on purpose, nonrandomly, from the Polish population of athletes. The basic criteria for selection were as follows: the voluntary will to participate in the study, the respondents had to be of senior age (between the ages of 20 and 29), and they had to have at least the second sports class. The remaining selection criteria were many years of sports experience, professional sports experience—three years and more, a current competition license, impeccable opinion of the coach, and documented sports achievements at various levels of competition (national, continental, world). The subjects were divided into equal samples (each *n* = 30) according to the trained sports discipline, and the following descriptive abbreviations were adopted: mountaineering, orienteering, biathlon, fitness, equestrian, canoeing, cycling, bodybuilding, athletics—long running, athletics—short runs, archery, alpine skiing, swimming, tobogganing, snowboarding, sport shooting, break dance, ballroom dancing, tennis, and sport climbing. Then, among all the surveyed competitors, athletes with significant international sports achievements were selected and qualified to the sample of champions (*n* = 56). The criterion for selecting nonrandom, purposeful respondents to the sample of champions was dictated by the recorded success (first, second, or third place) at international competitions in a given sports disciplines. This group includes medalists of the World Championship, European Championship, World Cup, European Cup, World Games 2017, and other prestigious international ranking tournaments. In the sample of champions, the following competitors were ranked: mountaineering (1), orienteering (3), biathlon (4), fitness (4), equestrian (1), canoeing (2), cycling (2), bodybuilding (4), athletics—long runs (8), athletics—short runs (2), archery (5), alpine skiing (3), swimming (3), tobogganing (3), snowboard (3), sport shooting (1), break dance (2), ballroom dancing (2), and sport climbing (3).

### 2.2 Method

A five‐factor personality model, known as the Big Five, was used to examine the athletes’ personality. A five‐factor personality model is made up of five measuring scales. They are marked with abbreviations derived from the names of the factors: neuroticism, extraversion, openness to experience, agreeableness, and conscientiousness. Neuroticism is a dimension that reflects emotional adjustment in relation to emotional imbalance, that is, emotionality in terms of negative emotions. Neuroticism means being prone to experiencing negative emotions such as fear, confusion, dissatisfaction, anger, guilt, and sensitivity to psychological stress. In turn, extraversion is a dimension that characterizes the quality and quantity of social interactions as well as the level of activity, energy, and the ability to feel positive emotions. Extroverted people are therefore friendly and talkative and willing to play and seek stimulation. They show optimism in life and a cheerful mood. Introverted people, on the other hand, are characterized by a lack of extrovert behavior rather than the complete opposite. Therefore, they show reserve in social contacts rather than hostility, lack of optimism, which does not necessarily mean pessimism or a lack of happiness, and a preference for being alone and shyness, which does not stand for social anxiety. Openness to experience is a dimension that describes an individual's tendency to seek and positively evaluate life experiences, tolerance toward novelty, and cognitive curiosity. People with high openness are interested in the phenomena of both the external and internal world and have a richer life in terms of the number of experiences. People with low openness are conventional in their behavior and conservative in views. And agreeableness is a dimension that describes a positive or negative attitude toward other people, an interpersonal orientation manifested in altruism in relation to antagonisms experienced in feelings, thoughts, and actions. At the cognitive level, this trait manifests itself as either trusting others or a lack of trust; on an emotional level—as sensitivity or indifference to other people's affairs; and on the behavioral level—as a cooperative attitude as opposed to a competitive one. Finally, conscientiousness is a dimension that characterizes the degree of organization, persistence, and motivation of an individual in goal‐oriented activities and describes a person's relationship to work.

The NEO—Five‐Factor Inventory (NEO‐FFI) tool was used to conduct the research due to the positioning of NEO‐FFI in the theoretical and methodological model compared with other approaches developed within the five‐factor model of personality; good psychometric characteristics; rich factual documentation of the measurement accuracy for the original version factors, which allows us to assume that the inventory may be useful in scientific and practical research; and acceptable timespan for the athletes to work with the questionnaire.

The NEO‐FFI questionnaire consists of 60 self‐report statements, the truthfulness of which in relation to themselves was assessed by the respondents on a five‐point scale: 1—“definitely not”, 2—“rather not”, 3—“I have no opinion”, 4—“rather yes”, and 5—“definitely yes”. The NEO‐FFI questionnaire has sten norms for 5 age‐groups (15–19, 20–29, 30–39, 40–49, 50–80), developed separately for women and men on the basis of large population samples. In addition, it is internally compatible. Its relevance was demonstrated on the basis of research on the relationship between the questionnaire results and the assessments of respondents made by observers, the heritability of the measured traits, and their correlation with other dimensions of personality and temperament. Factor validity was also verified. The results allow for a full description of the respondents’ personalities in terms of the Big Five and for forecasting their adaptation possibilities to the professional environment (Costa & McCrae, 2007).

Statistical analyses were performed using IBM SPSS Statistics, version 25. A series of one‐way analyses of variance were performed. For some measurements, Welch's correction for heterogeneity of variance was used. Parameters were estimated using the bootstrapping method with sampling set at 5,000 and 95% confidence intervals. When the assumption of homogeneity of variance was broken, Games–Howell tests were used in post hoc analyses. And if the assumption of homogeneity of variance was met, Tukey's tests were used. Additionally, due to the multiple comparisons made within each sport category, it was decided to adopt the Bonferroni correction for the significance level. In each sports category, 5 one‐way analyses of variance were performed, and the level of statistical significance for the analyses of variance was calculated as α = .01.

Then, the differences between athletes with significant sports successes (hereinafter referred to as “champions”) and other individual sportsmen were examined. For this purpose, a series of Student's *t* tests for independent samples were performed using a bootstrapping method set at 10,000 samples and a 95% confidence interval. In each sports group, 5 Student's *t* tests were performed, and the new level of statistical significance for the analyses of variance was calculated as α = .01.

### 2.3 Procedure

Each tested athlete agreed to participate in the research after getting acquainted with the information on its objectives and principles, expected effects, and possible benefits for the study participants. The respondents also familiarized themselves with the risk associated with undergoing the study, indicating the mode and the possibility of withdrawing from participation in the study at any stage. Moreover, the respondents were informed that they could ask questions and obtain answers to them. All respondents consented to the processing of data related to their participation in the research by the person conducting the research. The tests were carried out in rooms insulated from noise. The respondents had an hour to respond to the statements of the NEO‐FFI personality questionnaire. The research was carried out in groups of up to 30 people. After the research work was completed, the participants’ data were coded.

All procedures carried out in the human trials were in accordance with the ethical standards of the institution and the national research commission and the 1964 Helsinki Declaration and its subsequent amendments. The project received a positive opinion (number 20/2019) from the Senate Committee on Ethics of Scientific Research at the University School of Physical Education in Wrocław.

## 3 RESULTS

The results of analyses of individual sports groups showed statistically significant differences between sports disciplines for all personality traits. The strongest effect was observed for neuroticism, where the group differences accounted for approximately 29% of the variance in this trait. The second‐largest effect was observed in openness to experience—around 16% of the explained variability. The remaining effects ranged from 8% to 11% of the explained variance.

The lowest levels of neuroticism were observed in sports climbers, mountaineers, and tobogganers, respectively. The differences between these groups were not statistically significant. On the other hand, the athletes of the three abovementioned disciplines had a significantly lower level of neuroticism than other athletes. Additionally, alpine skiers and swimmers had significantly lower levels of neuroticism than equestrian, sport shooting, and ballroom dancing athletes. In addition, sport shooters had significantly lower neuroticism than cyclists.

The post hoc results in the extraversion dimension showed that swimmers had significantly lower levels of extraversion than climbers, alpine skiers, short‐distance runners, and tobogganers. Mountaineers additionally showed a significantly higher level of extraversion than sport shooters and break dance dancers. In addition, break dance dancers also recorded a lower level of extroversion in relation to canoeists and alpine skiers. In turn, alpine skiers showed a significantly higher level of extraversion than sport shooters.

The highest openness to experience was characteristic for mountaineers, and it was significantly higher than orienteering runners, biathletes, riders, canoeists, cyclists, bodybuilders, long‐distance runners, archers, alpine skiers, swimmers, tobogganers, ballroom dancers, and break dance dancers. On the other hand, break dance dancers were characterized by the lowest level of openness to experience, which was significantly different from the level of athletes of all disciplines except biathletes, riders, and swimmers. Swimmers also had a lower marker of openness to experience than alpine skiers, short‐distance runners, archers, tobogganers, snowboarders, sport shooters, ballroom dancers, tennis players, and sport climbers.

In the case of agreeableness, the lowest level was recorded by break dance dancers. In addition, it was statistically significantly lower than the level of agreeableness of athletes of all disciplines except biathletes, archers, swimmers, and sport shooters. Additionally, swimmers had a significantly lower level of agreeableness than sports climbers and snowboarders.

**FIGURE 1:**
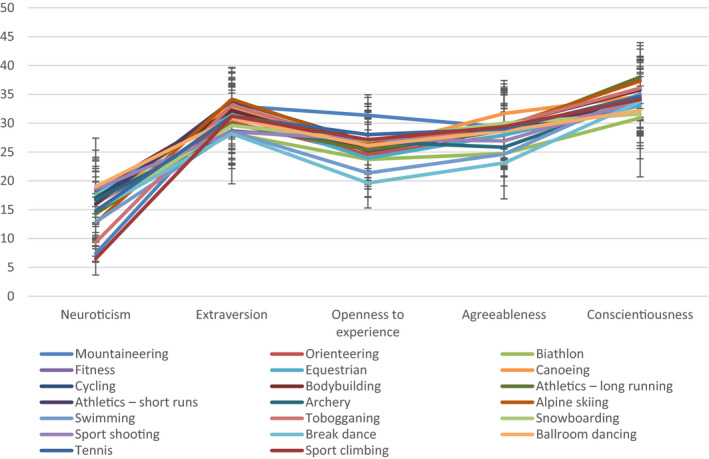
Pictured personality profiles of athletes of individual sports

In the post hoc conscientiousness analysis, only one statistically significant difference was found, which showed that mountaineers had a significantly higher level of conscientiousness than break dance dancers. The exact values of the parameters of the carried out analyses of variance are included in Table 1, and the whole is illustrated in Figure 1.

**TABLE 1:** Analysis of differences between the discussed sports disciplines for respective personality traits—one‐way analysis of variance

Disciplines	Personality traits									
	Neuroticism		Extraversion		Openness to experience		Agreeableness		Conscientiousness	
	*M*	*SD*	*M*	*SD*	*M*	*SD*	*M*	*SD*	*M*	*SD*
Mountaineering (*n* = 30)	7.40	1.35	33.03	3.64	31.37	3.55	29.30	5.69	37.50	4.10
Orienteering (*n* = 30)	16.10	6.06	30.20	7.30	24.60	5.20	27.90	5.28	34.33	6.16
Biathlon (*n* = 30)	17.33	6.77	28.20	8.72	23.73	5.11	24.73	7.86	30.90	10.21
Fitness (*n* = 30)	16.07	7.43	29.67	6.59	26.97	7.48	29.60	7.20	36.07	6.77
Equestrian (*n* = 30)	18.00	3.69	31.60	5.20	23.93	6.79	27.97	3.98	33.03	5.06
Canoeing (*n* = 30)	15.87	4.82	32.97	5.71	25.53	5.44	31.67	5.74	34.67	6.30
Cycling (*n* = 30)	17.03	4.57	32.27	6.62	25.20	6.14	28.70	6.57	34.27	6.49
Bodybuilding (*n* = 30)	18.40	9.02	32.17	6.53	25.67	5.13	29.67	6.14	35.77	5.28
Athletics—long running (*n* = 30)	14.30	7.35	29.83	5.90	25.37	6.80	28.83	6.57	38.00	5.44
Athletics—short runs (*n* = 30)	15.97	6.56	33.87	5.67	26.73	6.64	29.10	6.55	33.77	7.68
Archery (*n* = 30)	16.73	7.05	28.67	6.55	26.80	5.09	25.83	6.70	34.10	5.44
Alpine skiing (*n* = 30)	12.70	4.48	34.17	5.50	26.30	5.73	29.43	6.48	37.33	6.66
Swimming (*n* = 30)	12.90	2.64	28.47	3.55	21.37	4.11	24.63	3.93	35.13	4.73
Tobogganing (*n* = 30)	9.33	3.42	33.17	5.58	25.97	4.23	29.67	6.14	36.07	5.02
Snowboarding (*n* = 30)	14.90	5.01	29.60	6.86	26.97	6.45	30.00	6.27	31.60	7.76
Sport shooting (*n* = 30)	18.57	4.88	28.50	5.49	27.20	5.05	26.93	6.07	33.83	4.71
Break dance (*n* = 30)	14.93	2.86	28.17	2.77	19.63	4.33	23.10	2.41	33.73	2.73
Ballroom dancing (*n* = 30)	19.00	6.32	30.80	7.14	26.13	4.99	28.63	4.80	32.20	6.61
Tennis (*n* = 30)	14.77	5.05	31.20	5.14	28.03	5.26	29.00	7.02	34.67	6.10
Sport climbing (*n* = 30)	6.50	2.84	31.23	6.48	27.13	5.79	29.40	5.49	34.10	7.48
*F*	41.35^a^		4.88^a^		9.57^a^		8.24^a^		2.85^a^	
*df*	19; 211.35		19; 213.37		19; 213.59		19; 213.22		19; 213.36	
*p*	**<.001**		**<.001**		**<.001**		**<.001**		**<.001**	
*η* ^2^	0.29		0.10		0.16		0.11		0.08	

^a^ Correction for heterogeneity of variance.

**FIGURE 2:**
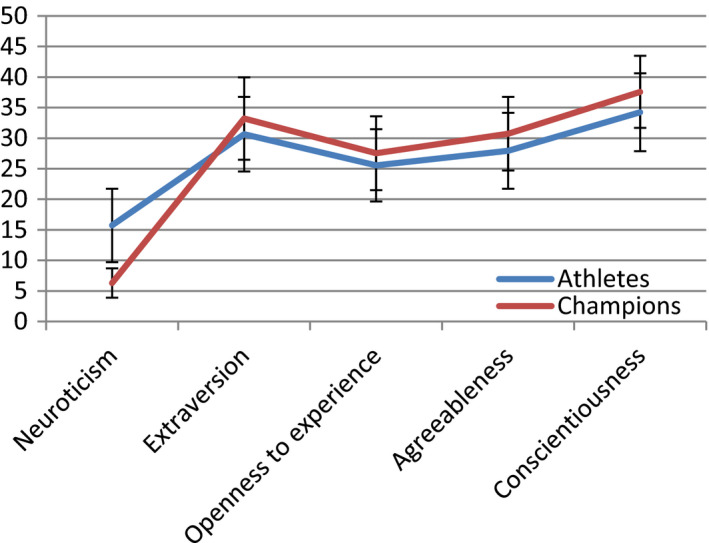
Pictured personality profiles of champions and other athletes of individual sports

Then, the results showed significant differences between the individual sports champions and other athletes in neuroticism, extraversion, agreeableness, and conscientiousness. A very strong effect was observed for neuroticism, a moderate effect for conscientiousness, and a weak effect (0.3 < d < 0.5) for extraversion and agreeableness. Similar to the above results, individual sports champions were characterized by a lower level of neuroticism, a higher level of extraversion, agreeableness, and conscientiousness than the rest of the athletes. Detailed results of the performed tests are presented in Table 2 and illustrated in Figure 2.

**TABLE 2:** Analysis of differences between champions and other athletes in the intensity of individual personality traits—Student's *t* tests

Variables	Athletes (*n* = 544)		Champions (*n* = 56)		*t*	*p*	Cohen's *d*
	*M*	*SD*	*M*	*SD*			
Neuroticism^a^	15.72	6.01	6.29	2.39	22.96	**<.001**	1.63
Extraversion	30.65	6.11	33.21	6.73	−2.96	.**008**	0.42
Openness to experience	25.55	5.92	27.54	6.04	−2.39	.020	0.34
Agreeableness	27.94	6.20	30.73	6.03	−3.21	.**001**	0.45
Conscientiousness	34.24	6.37	37.57	5.90	−3.75	**<.001**	0.53

^a^ Correction for heterogeneity of variance.

## 4 DISCUSSION

The obtained results showed that there are differences in the intensity of individual personality traits between the individual sports disciplines. There were statistically significant differences between sports disciplines in all personality traits included in the Big Five model. This indicates the existence of differences in the personality of athletes depending on the trained sports discipline. The obtained data show that sport influences the shaping of the personality of the assessed athletes, and it is confirmed by reports of other authors. One should take into account the differences between athletes due to the trained sports discipline (Chirivella & Martinez, 1994; Clingman & Hiliard, 1987; Kajtna et al., 2004; McEwan et al., 2019; McGill et al., 1986; Piepiora et al., 2018; Tok, 2013). Sports activity shapes the personality, and the formed personality traits have an impact on taking solutions in the starting situation. It should be associated with the specificity of sports competition and slightly different psychological requirements that sports disciplines impose on competitors. Therefore, there were significant differences among individual sports athletes in all five personality factors. In individual sports, unlike in combat sports and team sports, there is no direct contact with the opponent. The pressure from the opponent occurs indirectly (Piepiora, 2019; Piepiora & Witkowski, 2018, 2020a, 2020b). The measure of individual athletes’ performance, depending on the specificity of the discipline, is time, distance, height, etc. Hence, the supposition that the differences in all personality traits were revealed primarily in the divergent thinking and creativity of individual athletes (Costa & McCrae, 2007).

In addition, the results showed that individual sports champions had lower levels of neuroticism, higher levels of extroversion, agreeableness, and conscientiousness than the rest of the athletes. The general profile of athletes in terms of the Big Five is low neuroticism, high extraversion, and conscientiousness, as well as average openness to experience and agreeableness. From the obtained data, one could observe that individual sports champions were not only distinguished from the rest of the athletes by openness to experience (Anghel et al., 2009; McKelvie et al., 2003). These results are confirmed by research in the field of sports psychology (Binboga et al., 2012; Fuller, 2011; Ilyasi & Salehian, 2011; Kim et al., 2018; Litwiniuk et al., 2019; Mirzaei et al., 2013; Piedmont et al., 1999; Piepiora & Petecka, 2020; Piepiora & Witkowski, 2020a; Piepiora et al., 2018; Schutte et al., 2003; Shrivastaval et al., 2010) and indicate that the intensity level of an athlete's personality traits can be a predictor of a sports score. Therefore, personality traits should be used as predictors of sports performance. On this basis, it can be presumed that the level of intensity of neuroticism, extraversion, agreeableness, and conscientiousness may determine success in individual sports. On the other hand, the level of openness to experience, as in the case of comparisons of sports, depends on the specificity of the trained sport.

The obtained research results partially confirmed the findings of Allen et al., (2011) that the personality determinants of success in individual sports are low neuroticism, high extraversion, and high openness to experiences. Namely, the significance of low neuroticism was confirmed, which was also demonstrated by Piepiora and Witkowski (2020b); the significance of high extraversion was confirmed, as also stated by Allen et al., (2021); the importance of high openness to experience was however negated, and finally, the dimensions of high agreeableness and high conscientiousness were demonstrated.

Here, the strengths and limitations of the research should be equally indicated. The research sample was homogeneous in terms of ethnicity, gender, and the age range of 20–29 years. Athletes of other nationalities, women, and other age‐groups were not included. The research was conducted on a large group of respondents from individual sports disciplines that are popular in Poland. However, it was not possible to examine the players from all of the individual sports disciplines trained in Poland. The variables were distributed in equal samples. The group of champions included Polish winners of individual sports disciplines with international successes. Therefore, the obtained research results can only be applied to a specific population of athletes. The author of his research results does not question the current research results, but only supplements the existing knowledge about personality in sport in the twenty‐first century. The obtained research results suggest that the five‐factor model of personality may help to distinguish the mental levels of sport involvement and may help to identify tactical actions in individual sports.

As there is no prior knowledge about the personality of the surveyed athletes from the earlier periods of their sports careers, there is no basis for concluding how many years of sports training had an impact on possible modifications of this important human property. Moreover, it is not known to what extent the specificity of trained sports disciplines and coaching could have had a decisive influence on shaping the personality of athletes, apart from the influence of the coach and other entities from the players’ closest social environment. Therefore, further research should take into account social and cultural factors.

## 5 CONCLUSIONS

There are differences in the personalities of athletes depending on the trained individual sports discipline. Each sports discipline is characterized by slightly different psychological requirements for athletes. The undertaken sports activity shapes the personality. And the shaped personality traits have an impact on solutions taken in the starting situation.

Individual sports champions are not only distinguished from other athletes by their openness to experience. Therefore, the level of intensity of neuroticism, extroversion, agreeableness, and conscientiousness may determine the result in individual sports competition. And the intensity level of openness to experiences, as in the case of comparisons of sports, depends on the specificity of sports competition.

## CONFLICT OF INTEREST

None declared.

### PEER REVIEW

The peer review history for this article is available at https://publons.com/publon/10.1002/brb3.2145.

## Data Availability

The data that support the findings of this study are available on request from the corresponding author. The data are not publicly available due to privacy or ethical restrictions.
